# Fatty Acid and Peptide Profiles in Plasma Membrane and Membrane Rafts of PUFA Supplemented RAW264.7 Macrophages

**DOI:** 10.1371/journal.pone.0024066

**Published:** 2011-08-24

**Authors:** Julia Schumann, Alexander Leichtle, Joachim Thiery, Herbert Fuhrmann

**Affiliations:** 1 Faculty of Veterinary Medicine; Institute of Physiological Chemistry, University of Leipzig, Leipzig, Germany; 2 Faculty of Medicine; Institute of Laboratory Medicine, Clinical Chemistry and Molecular Diagnostics, University of Leipzig, Leipzig, Germany; Cornell University, United States of America

## Abstract

The eukaryotic cell membrane possesses numerous complex functions, which are essential for life. At this, the composition and the structure of the lipid bilayer are of particular importance. Polyunsaturated fatty acids may modulate the physical properties of biological membranes via alteration of membrane lipid composition affecting numerous physiological processes, e.g. in the immune system. In this systematic study we present fatty acid and peptide profiles of cell membrane and membrane rafts of murine macrophages that have been supplemented with saturated fatty acids as well as PUFAs from the n-3, the n-6 and the n-9 family. Using fatty acid composition analysis and mass spectrometry-based peptidome profiling we found that PUFAs from both the n-3 and the n-6 family have an impact on lipid and protein composition of plasma membrane and membrane rafts in a similar manner. In addition, we found a relation between the number of bis-allyl-methylene positions of the PUFA added and the unsaturation index of plasma membrane as well as membrane rafts of supplemented cells. With regard to the proposed significance of lipid microdomains for disease development and treatment our study will help to achieve a targeted dietary modulation of immune cell lipid bilayers.

## Introduction

In the last decade the traditional fluid mosaic model of the plasma membrane as introduced 1972 by Singer and Nicholson [1] has been extended to the lipid raft hypothesis [2]. According to this structurally and functionally distinct domains can be distinguished within the cell membrane due to their specific lipid compositions [2]. In a Keystone Symposium on Lipid Rafts and Cell Function the resulting microenvironments were defined as “small (10–200 nm), heterogeneous, highly dynamic, sterol- and sphingolipid-enriched domains that compartmentalize cellular processes” [3]. Small rafts were proposed to be stabilized via protein-protein as well as protein-lipid interactions. [3]. A variety of proteins have been shown to be enriched in membrane rafts including GPI-anchored proteins, flotillin, receptor tyrosine kinases and G protein-coupled receptors [4]. Taking the dynamic nature of membrane domains into account the rafts have been supposed to scaffold certain signaling molecules while excluding others and thus function as a unique signaling platform [5]. In fact, raft protein interaction is discussed to be central to biological information transmission [6]. Further cellular processes membrane domains have been implicated include membrane trafficking and molecular sorting [7].

A range of macrophage functions interrelate with membrane rafts. This includes endotoxin-mediated activation, phagocytosis and MHCII-mediated antigen presentation [8], [9]. Moreover, the domains have been identified to play an important role in infection biology [10]. It has been shown that a wide variety of pathogens target rafts to invade host cells [10]. Likewise, bacterial toxins have been demonstrated to enter cells via certain toxin-associated receptors which are concentrated in membrane rafts [11]. Stimulation of RAW264.7 macrophages with LPS has been described to result in a selective recruitment and activation of proteins, e.g. proteasome subunits, in the membrane rafts [12].

Polyunsaturated fatty acids (PUFAs) are known to affect numerous physiological processes being implicated in cardiovascular, neurological and immune function as well as cancer [13], [14]. Due to their pleiotropic effects on mammalian physiology a base mode of action common to all cells has been postulated [14]. This functional link may be the modulation of the physical properties of biological membranes via alteration of membrane lipid composition [14]. In fact, changes in the fatty acid composition of immune cell membranes have been shown to exert impact on phagocytosis, T cell signaling as well as antigen presentation [15]. Due to the significance of lipid interactions for the formation of membrane domains, PUFAs have been speculated to perturb structure, organization and function of rafts [11]. Moreover, domain structure and composition have been hypothesized to directly reflect biochemical and physiological processes [16]. Besides, studies concerning the effects of PUFAs from the n-3 family, as eicosapentaenoic acid (EPA) and docosahexaenoic acid (DHA), on immune cell function proposed a selective displacement of acylated proteins from membrane rafts by virtue of a modified raft lipid environment [11].

In this systematic study we present fatty acid and peptide profiles of plasma membrane and rafts of macrophages from the murine cell line RAW264.7 that have been supplemented with saturated fatty acids as well as PUFAs from the n-3, the n-6 and the n-9 family.

## Results

### Fatty acid composition in plasma membrane and membrane rafts of RAW264.7 cultured in basic medium

Distinct differences in the fatty acid composition of plasma membrane and membrane rafts from RAW264.7 cultured in basic medium could be seen. In the plasma membrane the distribution of the fatty acid families was 2.5% n-3, 5.6% n-6, 27.0% n-7, 28.7% n-9 and 36.2% saturated (Table 1). In the rafts the proportion of the fatty acid families was as follows: 0.2% n-3, 3.3% n-6, 24.7% n-7, 50.0% n-9, 21.8% saturated (Table 2). Of note, the only detectable n-3 fatty acid in the membrane rafts was C20∶5n3 (EPA). The differences in the fatty acid profiles of the two membrane domains were also reflected by the Methylene Bridge Index (MBI) which was about 3 times greater for plasma membrane than for membrane rafts (Table 1, Table 2). Fatty acids identified to be abundant in both plasma membrane and membrane rafts were C18∶1n9 (oleic acid), C18∶1n7 (vaccenic acid), C16∶1n7 (palmitoleic acid), C18∶0 (stearic acid), C16∶0 (palmitic acid) and C14∶0 (myristic acid) (Table 3).

**Table 1:** Fatty acid patterns [nmol/mg protein] and MBI in plasma membrane of PUFA supplemented cells.

	Basic medium	LNA	EPA	DHA	LA	γ-LNA	AA
**n-3**	53.4±17.1^a^	324.0±220.9^a^	388.3±20.4^c^	231.2±16.8^b^	48.2±11.1^a^	33.1±4.2^a^	42.5±21.1^a^
**n-6**	119.4±39.9^a^	113.2±62.9^a^	77.2±6.8^a^	55.1±4.4^b^	302.9±40.8^c^	251.3±38.2^c^	315.8±39.8^c^
**n-7**	572.7±68.3^a^	415.1±132.5^a^	531.9±21.4^a^	355.9±19.6^b^	413.8±60.4^b^	360.0±53.0^b^	319.4±55.5^b^
**n-9**	607.4±218.6^a^	504.9±83.8^a^	276.9±68.0^b^	164.1±12.5^c^	375.2±95.0^a^	208.9±15.2^b^	467.6±190.5^a^
**saturated**	766.3±130.2^a^	764.4±125.6^a^	1136.0±63.4^b^	514.3±54.8^c^	698.7±90.2^a^	719.5±280.7^a^	722.4±362.7^a^
**MBI**	21.3±5.2^a^	63.5±36.2^a^	74.0±5.0^c^	93.5±4.9^d^	44.0±4.1^b^	49.6±11.2^b^	64.6±14.9^b^

Fatty acid patterns [nmol/mg protein] and Methylene Bridge Index (MBI) in plasma membrane of RAW264.7 cultured for 72h in basic medium as well as in medium supplemented with 15 µmol/l alpha-linolenic acid (LNA), eicosapentaenoic acid (EPA), docosahexaenoic acid (DHA), linoleic acid (LA), gamma-linolenic acid (γ-LNA) and arachidonic acid (AA) respectively. Data are mean±S.D. (n = 6). Superscript letters across a row denote significant differences.

**Table 2:** Fatty acid patterns [nmol/mg protein] and MBI in membrane rafts of PUFA supplemented cells.

	Basic medium	LNA	EPA	DHA	LA	γ-LNA	AA
**n-3**	4.3±10.5^a^	279.0±318.1^a^	200.6±39.5^b^	154.2±9.2^b^	n.d.	n.d.	n.d.
**n-6**	79.6±9.6^a^	135.0±73.5^a^	79.0±11.3^a^	89.0±34.0^a^	251.3±116.2^b^	179.2±37.0^b^	196.1±45.0^b^
**n-7**	604.7±81.2^a^	382.9±121.6^b^	425.6±98.4^b^	303.8±41.6^b^	419.4±130.0^a^	209.9±53.0^c^	264.0±44.3^b^
**n-9**	1224.0±505.7^a^	1403.0±500.6^a^	480.0±41.8^b^	407.5±240.0^b^	1089.0±133.8^a^	553.3±276.3^a^	1458.0±169.0^a^
**saturated**	534.4±52.4^a^	402.9±220.9^a^	818.4±349.4^a^	388.1±482.0^a^	385.6±139.8^a^	100.6±34.0^c^	251.6±64.9^b^
**MBI**	6.7±5.8^a^	44.6±42.0^a^	50.0±11.1^bc^	78.4±35.4^c^	20.6±8.2^b^	46.8±28.1^b^	23.4±5.5^b^

Fatty acid patterns [nmol/mg protein] and Methylene Bridge Index (MBI) in membrane rafts of RAW264.7 cultured for 72h in basic medium as well as in medium supplemented with 15 µmol/l alpha-linolenic acid (LNA), eicosapentaenoic acid (EPA), docosahexaenoic acid (DHA), linoleic acid (LA), gamma-linolenic acid (γ-LNA) and arachidonic acid (AA) respectively. Data are mean±S.D. (n = 6). Superscript letters across a row denote significant differences. n.d.  = below detection limit.

**Table 3:** Fatty acids [nmol/mg protein] dominating in plasma membrane and membrane rafts of cells cultured in basic medium.

	Plasma membrane	Membrane rafts
**C18∶1n9**	1115.0±498.6^a^	506.5±205.0^a^
**C18∶1n7**	377.4±74.4^a^	331.1±48.0^a^
**C16∶1n7**	217.3±11.4^a^	203.0±18.2^a^
**C18∶0**	128.9±39.5^a^	178.7±32.9^a^
**C16∶0**	156.3±41.0^a^	434.6±127.5^b^
**C14∶0**	126.4±11.5^a^	97.5±11.9^b^

Fatty acids [nmol/mg protein] dominating in plasma membrane and membrane rafts of RAW264.7 cultured for 72h in basic medium. Data are mean±S.D. (n = 6). Superscript letters across a row denote significant differences. C18∶1n9: oleic acid; C18∶1n7: vaccenic acid; C16∶1n7: palmitoleic acid; C18∶0: stearic acid; C16∶0: palmitic acid; C14∶0: myristic acid.

### Fatty acid composition of plasma membrane and membrane rafts of RAW264.6 cultured in PUFA supplemented medium

Supplementation of culture medium with the n-3 PUFA LNA (C18∶3n3), EPA (C20∶5n3) and DHA (C22∶6n3) respectively resulted in an increase in the content of n-3 fatty acids in both plasma membrane and membrane rafts (Table 1, Table 2). This rise was more pronounced for membrane rafts than for plasma membrane leading to an approaximation in n-3 PUFA between the two membrane domains (Table 1, Table 2). Fatty acids detected to be significantly enriched in both plasma membrane and membrane rafts were C22∶5n3 (docosapentaenoic acid) for LNA supplementation, C20∶5n3, C22∶5n3 and C22∶6n3 for EPA supplementation as well as C22∶6n3 for DHA supplementation (data not shown). Further n-3 fatty acids detectable in plasma membrane as well as membrane rafts of n-3 PUFA supplemented cells included C18∶3n3, C20∶3n3 (eicosatrienoic acid), C20∶4n3 (eicosatetraenoic acid), C20∶5n3, C22∶5n3 and C22∶6n3 (data not shown). Besides, there was a decrease in the content of n-7 and n-9 monounsaturated fatty acids (Table 1, Table 2) mainly C18∶1n7 (vaccenic acid) and C18∶1n9 (oleic acid). The enrichment in n-3 PUFA content was connected with a rise of the MBI of both membrane domains (Table 1, Table 2). Compared to RAW264.7 cultured in basic medium the calculated MBI of the plasma membrane increased by factor 3 (LNA supplementation) to 4.4 (DHA supplementation) (Table 1, Table 2). For membrane rafts an increase by factor 7.1 (LNA supplementation) to 12.4 (DHA supplementation) was observed (Table 1, Table 2).

Enrichment of the culture medium with PUFAs of the n-6 family (LA (C18∶2n6), γ-LNA (C18∶3n6), AA (C20∶4n6)) resulted in a rise in the content of n-6 fatty acids in plasma membrane as well as membrane rafts (Table 1, Table 2). For both membrane domains an increase of about 2.5 times could be detected (Table 1, Table 2). Significantly enriched fatty acids in plasma membrane and membrane rafts include C18∶2n6 and C20∶4n6 for LA supplementation, C18∶3n6, C20∶3n6 (dihomo-gamma-linolenic acid), C20∶4n6 and C22∶4n6 (docosatetratenoic acid) for γ-LNA supplementation as well as C20∶4n6 and C22∶4n6 for AA supplementation. In addition to the rise in n-6 PUFAs, a decrease in the content of n-7 and n-9 monounsaturated fatty acids (in particular C16∶1n7 (palmitoleic acid) and C18∶1n7 (vaccenic acid)) was observed in plasma membrane and membrane rafts (Table 1, Table 2). Furthermore, n-6 PUFA enrichment was connected with a significant increase in MBI of the cells (plasma membrane: 2.1 times with LA supplementation to 3.0 times with AA supplementation; membrane rafts: 3.1 times with LA supplementation to 7.0 times with γ-LNA supplementation; Table 1, Table 2).

Supplementation of the culture medium with the n-9 fatty acid oleic acid (C18∶1n9) as well as the saturated fatty acids palmitic acid (C16∶0) and stearic acid (C18∶0) resulted in only marginal modifications in the fatty acid composition of plasma membrane and membrane rafts (data not shown).

### Peptide profiles of plasma membrane and membrane rafts

Peptide profiles of plasma membrane and membrane rafts from RAW264.7 cultured in basic medium were found to resemble each other (Table 4). However, eight peaks with *m/z* 1165, 1390, 2378, 4286, 5008, 7771 and 9108 were solely detected in the plasma membrane (Table 4). For membrane rafts no such marker peaks could be identified.

**Table 4:** Peptide profiles of plasma membrane and membrane rafts.

Plasma membrane(*m/z*)	Membrane rafts(*m/z*)	Notions
1015	1015	Sensitive to fatty acid composition of membrane rafts
1043	1043	Sensitive to fatty acid composition of membrane rafts
1052	1052	
1059	1059	Sensitive to fatty acid composition of membrane rafts
1067	1067	
1073	1073	
1083	1083	
1087	1087	Sensitive to fatty acid composition of membrane rafts
1115	1115	Sensitive to fatty acid composition of membrane rafts
1131	1131	
1147	1147	
1165		Unique peak for plasma membrane
1190	1190	
1200	1200	
1263	1263	
1275	1275	
1280	1280	
1291	1291	
1310	1310	
1334	1334	
1385	1385	
1390		Unique peak for plasma membrane; Sensitive to fatty acid composition of plasma membrane
1408	1408	
1448	1448	
1461	1461	Sensitive to fatty acid composition of plasma membrane and membrane rafts
1511		Unique peak for plasma membrane
1516	1516	Sensitive to fatty acid composition of plasma membrane
1534	1534	
1552	1552	
1575	1575	Sensitive to fatty acid composition of plasma membrane
1618	1618	
2211	2211	
2378		Unique peak for plasma membrane
2868	2868	
2909	2909	
2995	2995	
3012	3012	
3035	3035	
3050	3050	
3066	3066	Sensitive to fatty acid composition of plasma membrane
3175	3175	
3192	3192	
3217	3217	
3779	3779	
3796	3796	
3820	3820	
3853	3853	
3906	3906	
3922	3922	
3947	3947	
3979	3979	
3996	3996	
4020	4020	
4286		Unique peak for plasma membrane
4583	4583	
4709	4709	
4725	4725	
4751	4751	
4968	4968	
4983	4983	
5008		Unique peak for plasma membrane
6116	6116	
6175	6175	
6214	6214	
6231	6231	
6256	6256	
6288	6288	
6304	6304	
6377	6377	
7112	7112	
7771		Unique peak for plasma membrane; Sensitive to fatty acid composition of plasma membrane
9108		Unique peak for plasma membrane

Peptide profiles of plasma membrane and membrane rafts of RAW264.7 cultured for 72h in basic medium. Peptidome separation was done using the CLINPROT profiling purification kit from Bruker Daltonics (Billerica, USA) by means of magnetic particles with magnetic bead-hydrophobic interaction (MB-HIC C8).

Supplementation of RAW264.7 with PUFA was found to have only slight effects on the peptide profiles of both plasma membrane and membrane rafts. In plasma membrane the peaks with *m/z* 1390, 1461, 1516, 1575, 3066 and 7771, and in membrane rafts peaks with *m/z* 1015, 1043, 1059, 1087, 1115 and 1461 turned out to be sensitive to the fatty acid composition of the membrane compartment (Table 4).

An *in silico* search against SwissProt database using the TagIdent tool from ExPASy Proteomics Server (error tolerance: 350 ppm, taxonomy: *Mus musculus*) did not result in a reliable identification of the peaks mentioned.

## Discussion

Eukaryotic cell plasma membrane not only forms a selective barrier controlling the transport of molecules inside and outside but also regulates cell communication. Further cellular processes the lipid bilayer is involved include proliferation, differentiation, secretion, migration, invasion and phagocytosis to name a few. Of note, the composition and the structure of the plasma membrane is known to play a key role in these processes [10].

According to our current state of knowledge lipids actively influence the dynamics and the protein organization of membranes [10]. In particular, lipids are of importance in the formation of membrane rafts. Membrane rafts are discussed as control points of plasma membrane dependent biological events such as signaling [5], [7], sorting and trafficking [7], [17], cell adhesion [18], migration [18], pathogen entry [10], [11], [19] and immune response [11], [20]. Moreover, rafts have been suggested to play critical roles in many human diseases [21]–[23]. Alterations in membrane lipid composition have been shown to shift raft structure and function [21], [22]. The dietary modulation of membrane rafts therefore is assumed as a promising treatment for membrane associated diseases [22], [24].

Supplementation of the culture medium with PUFAs from the n-3 and the n-6 family resulted in significant alterations in the fatty acid composition of both plasma membrane and membrane rafts of RAW264.7 macrophages. Of note, the PUFAs added to the culture medium were not only incorporated but also metabolized leading to a significant increase of their desaturation and elongation products. This was accompanied by a decrease in the content of monounsaturated fatty acids of the n-7 and the n-9 family in plasma membrane as well as in membrane rafts. The enhanced proportion of polyunsaturated fatty acids brought about an increase of the MBI. The MBI is defined as the mean number of bis-allyl-methylene positions per fatty acid contained in the membrane domains. It is calculated by multiplying the number of bis-allyl-methylene positions contained in each fatty acid methyl ester species by its respective mole fraction and summed for all fatty acids present [25]. The higher the MBI, the higher the un-saturation of the membrane lipids. Remarkably, depending on the fatty acid added both in plasma membrane and in membrane rafts there was a significant difference in MBI gained with DHA supplementation resulting in the highest and LA supplementation resulting in the lowest MBI. Thus, there is a relation between the number of methylene bridges of a PUFA supplemented and the dimension of un-saturation of cellular membranes. However, for all fatty acids tested the MBI of membrane rafts was lower compared to the MBI of plasma membrane.

So far, investigations concerning the effects of PUFA supplementation on membrane rafts lipid composition have been focused on the n-3 fatty acids EPA and DHA [5], [26]–[29]. At this, EPA and DHA have been identified to modify membrane rafts characterized by an enrichment of the PUFAs added and a decrease of monounsaturated fatty acids, which is connected with alterations in raft size, stability and distribution [5], [26]–[29]. Thus, our data are in accordance with these previous results. Moreover, our study provide evidence that these effects are not exclusive to long-chain polyunsaturated fatty acids of the n-3 family but are also true of shorter chain n-3 fatty acids such as LNA as well as for PUFAs from the n-6 family such as LA, γ-LNA and AA. Overall, these findings hint towards the impact of dietary fatty acids of both the n-3 and the n-6 family on the biochemical make up of lipid bilayers.

Noteworthy, the phagocytic activity of neutrophils and monocytes has been shown to depend on the membrane fatty acid composition [15]. Phagocytic capacity was found to be negatively correlated to saturated fatty acid content and to be positively correlated with unsaturated fatty acid content whether n-3 PUFAs or n-6 PUFAs [15]. This effect is discussed to be in part due to alterations in receptor expression, but primarily to origin in the physical nature of the membrane of the phagocytes [15]. The incompatibility between the highly flexible, very long chain PUFAs and the rigid sterol moiety of cholesterol is well known [14], [30]. In fact, a growing number of studies verify the tremendous impact of PUFAs on the physical properties of membranes. This includes membrane permeability [31], membrane elasticity and curvature strain [32]–[34] thereby modulating membrane fusion [35], vesicle formation [36], lateral lipid segregation [37] and flip-flop mechanisms [38].

Moreover, changes in membranes properties due to altered lipid composition are proposed to affect the organization and interactions between lipids and proteins [14]. PUFA incorporation into membrane lipids of a Jurkat T cell line as well as COS-1 cells has been shown to result in a selective displacement of the Src family kinases Lck, Fyn and LAT from membrane rafts [26], [39]–[41]. Further membrane-associated proteins known to be affected by PUFA enrichment of the lipid bilayer include the signaling proteins Ras, Akt and Her-2/neu [24] as well as the immunogenic receptors TLR4 [42], IL-2 and FcR [28], [43]. Treatment of antigen-presenting cells with EPA or DHA has been found to decrease MHCII expression and MHCII-dependent antigen presentation by the monocytes and dendritic cells *in vitro* [15]. Altogether, these data underline the importance of membrane lipid composition for cell communication and immune defense. Actually, two possible routes are discussed by which PUFA may interfere with protein targeting to membrane rafts. On the one hand the PUFAs have been shown to inhibit protein acylation with saturated acyl moieties [41]. On the other hand supplementation of cells with PUFA results in the aforementioned alteration in membrane lipid environment. Transmembrane as well as acylated proteins are believed to directly interact with cholesterol-sphingolipid complexes by means of their transmembrane amino acids and their saturated acyl moieties respectively [14], [44]. Changes in the lipid composition of the membrane therefore may have a profound impact on the micro domain localization of proteins.

To obtain deeper insights into the interactions between lipids and proteins we performed a peptide profiling analysis of plasma membrane and membrane rafts of PUFA supplemented RAW264.7 macrophages. Peptide profiling can be accomplished in a variety of biological samples, i.e. tissues, cells, organelles, blood samples and supramolecular complexes, such as lipid bilayers [45]. To minimize any sampling-related disturbances the samples were collected, stored and processed according to a feasible and highly standardized pre-analytic protocol [46]. The peptide profiles of plasma membrane and membrane rafts of RAW264.7 macrophages were found to highly resemble each other. Nevertheless, eight peaks unique for plasma membrane could be detected. Unfortunately, at present it was not possible to reliably identify the mentioned peaks by means of an *in silico* search in a leading protein database. The incorporation of PUFAs into the lipid bilayer was observed to have slight effects on the overall peptide profile of both plasma membrane and membrane rafts of RAW264.7 macrophages. Indeed, several peaks sensitive to the fatty acid composition of both plasma membrane and membrane rafts could be seen. The ability of PUFAs to influence the lateral organization of membrane micro domains *in situ*, however, remains unclear.

Taking together, using the murine macrophage cell line RAW264.7 as a model system we identified PUFAs from both the n-3 and the n-6 family to impact lipid and protein composition of plasma membrane and membrane rafts in a similar manner. In addition, we found a relation between the number of bis-allyl-methylene positions of the PUFA added and the un-saturation index of plasma membrane as well as membrane rafts of supplemented cells. The concentrations of free fatty acids tested in our study match physiological conditions [47], and the membrane preparation was performed detergent-free thus underlining the relevance of the gained results. With regard to the proposed significance of lipid micro domains for disease development and treatment our systematic study will help to understand dietary modulation of immune cell lipid bilayers.

## Materials and Methods

### Materials

All chemicals and reagents were obtained from Sigma-Aldrich (Taufkirchen, Germany) unless noted otherwise. Cell culture flasks were purchased from Greiner Bio-One (Frickenhausen, Germany). HEPES (25 mmol/L)-buffered RPMI 1640 culture medium containing 300 mg/L L-glutamine was acquired from PAA Laboratories GmbH (Cölbe, Germany).

### Cell culture

The permanent mouse monocyte/macrophage cell line RAW264.7 (ATCC number TIB-71) was used. The RAW264.7 cells were cultured in RPMI 1640 medium supplemented with 4.5 g/L glucose and 5% FCS (basic medium). The fatty acids alpha-linolenic acid (LNA), eicosapentaenoic acid (EPA), docosahexaenoic acid (DHA), linoleic acid (LA), gamma-linolenic acid (γ-LNA), arachidonic acid (AA), oleic acid, palmitic acid or stearic acid (all Biotrend, Köln, Germany) were included in the culture medium in concentrations of 15 µmol/L using ethanol as a vehicle (0.2% v/v final ethanol concentration). Cells were incubated in 75 cm^2^ cell culture flasks at a density of 1×10^6^ cells/mL for 72 h at 37°C and 5% CO_2_ in a humidified atmosphere.

### Detergent-free preparation of membrane rafts and plasma membrane

Preparation of membrane rafts and plasma membrane was carried out detergent-free per density gradient ultracentrifugation by the method introduced by Macdonald and Pike [48] with slight modifications [49]. 2×10^7^ cells were suspended in isolation buffer (20 mM Tris-HCl, pH 7.8, 250 mM sucrose, 1 mM CaCl_2_, 1 mM MgCl_2_, supplemented with 0.2% protease inhibitor cocktail set III (Calbiochem, Darmstadt, Germany)) and homogenized by 20 passages through a 22G syringe needle. After centrifugation (1000 g for 10 min) aspirated supernatants were mixed with an equal volume (2 mL) of separating buffer (120 mM Tris-HCl, pH 7.8, 250 mM sucrose containing 50% OptiPrep) and placed in the bottom of a 12 mL centrifuge tube. An 8 mL gradient of 0% to 20% OptiPrep was poured on top of the lysate. Gradients were centrifuged for 90 min at 52000 g using an SW-41 rotor (Beckman Coulter Incorporation, Fullerton, USA) by means of a Sorvall Discovery 90 SE ultracentrifuge (Hitachi, Tokio, Japan). Subsequently, gradients were fractionated into 12 1 mL fractions from the top to the bottom. Total protein in each fraction was determined using the Roti^®^Quant assay kit (Roth, Karlsruhe, Germany). Fractions were analyzed by Western blot for the presence of the raft marker EGF receptor [4], [48], [50]–[52] (using anti-EGFR antibody, Biolegend, Diego, USA) as well as the plasma membrane marker transferrin receptor [8], [12], [48] (using anti-TFRC antibody, GeneTex, Irvine, USA). EGF receptor was found to be enriched in fraction 1 ( =  membrane raft fraction), transferrin receptor was found to be enriched in fraction 7 ( =  plasma membrane fraction).

### Fatty acid composition analysis

Fatty acid composition was analyzed by lipid extraction and subsequently gas chromatography [53]. The membrane lipids were trans-esterified with 500 µL methanolic HCl, 250 µL n-hexane and 500 µL internal standard (0.8 mg Di-C17-phosphatidylcholine in 1 mL methanol with 0.2% Butylhydroxytoluol as antioxidant). After cooling-off, 500 µL n-hexane and 1 mL aqua dest. were added. The upper hexane phase was evaporated with nitrogen. The fatty acid methylesters (FAME) were taken up in 60 µL n-hexane. An aliquot of 1 µL was injected on-column on a Varian CP 3800 gas chromatograph (Varian, Darmstadt, Germany) equipped with an Omegawax TM 320 column (0.32 mm internal diameter, 30 m length) (Supelco, Bellefonte, USA). The column temperature was 200°C. Based on the ratio of a fatty acid (weight %) detected by gas chromatography and the number of its bis-allyl-methylene positions the Methylene Bridge Index (MBI) was calculated [25].

### Peptidome profiling

The isolation, concentration and purification of peptides from the prepared samples of membrane rafts and plasma membrane was done using the Profiling kit MB-HIC 8 (Bruker Daltonics, Billerica, USA) by means of magnetic particles with defined surface functionalities (magnetic bead-hydrophobic interaction). The workflow of the peptidome separation procedure includes the binding of the sample to the magnetic beads, the washing of the sample bound to the magnetic beads and the elution of the sample bound to the magnetic beads. The magnetic bead preparation was processed in an automated one-step procedure by the CLINPROT liquid handling robot (Bruker Daltonics, Billerica, USA) according to the manufactures instructions. A linear MALDI-TOF mass spectrometer (Autoflex I, Bruker Daltonics, Billerica, USA) was used for the peptidome profiling. Mass calibration was done using the standard calibration mixture of peptides and proteins in a mass range of 1 to 10 kDa. Mass spectra were recorded and processed using the AutoXecute tool of the flexControl acquisition software (version 2.0; Bruker Daltonics, Billerica, USA). Each sample was processed 4-fold to improve data reproducibility. Bioinformatic data analysis was performed by means of the ClinPro Tools (CPT) 2.0.365 Software (Bruker Daltonics, Billerica, USA). The workflow started by loading of two selected classes (e.g. unsupplemented and supplemented plasma membrane fractions, unsupplemented and supplemented membrane rafts fractions, plasma membrane and membrane rafts fractions of equally supplemented cells). The software package used includes an automated raw data pretreatment workflow, comprising baseline subtraction with 80% baseline flatness (Convex Hull), normalization of spectra according to the total ion count, an alignment of peaks with a signal-to-noise ratio (S/N) >3 to prominent peaks with S/N >100 and a peak-picking procedure resulting in peaks defined as dynamic *m/z* ranges. Savitzky-Golay smoothing was deactivated to avoid the blurring of peaks (CPT manual version 2.0). Peak statistics were done using 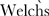 *t* test without multiple testing correction. Finally, the software provides a list of peaks sorted along the statistical difference between two classes [46].

### Statistical analysis

Data are shown as means ± standard deviation (S.D.). Two-way analysis of variance followed by unpaired Students *t* test was used to identify significant differences between means. The statistical analysis was carried out by means of the program GraphPad Prism 4 (GaphPad Software, La Jolla, USA). In all cases, *p*<0.01 was assumed to indicate significant differences.
